# The Adaptive Lab Mentor (ALM): An AI-Driven IoT Framework for Real-Time Personalized Guidance in Hands-On Engineering Education

**DOI:** 10.3390/s25247688

**Published:** 2025-12-18

**Authors:** Md Shakib Hasan, Awais Ahmed, Nouman Rasool, MST Mosaddeka Naher Jabe, Xiaoyang Zeng, Farman Ali Pirzado

**Affiliations:** 1 School of Electronic Information Engineering, China West Normal University, Nanchong 637002, China; shakib@mails.ccnu.edu.cn (M.S.H.);; 2 School of Computer Science, China West Normal University, Nanchong 637002, China; jabe@stu.cwnu.edu.cn; 3 School of Computer Science and Engineering, University of Electronic Science and Technology of China—UESTC, Chengdu 611731, China; 202011081605@std.uestc.edu.cn; 4 Tecnológico de Monterrey, School of Engineering and Sciences Monterrey, Monterrey 64849, Mexico; a00836551@tec.mx

**Keywords:** adaptive learning, intelligent tutoring systems, IoT in education, sensors data analytics, experiential learning

## Abstract

Engineering education is based on experiential learning, but the problem is that in laboratory conditions, it is difficult to give feedback to the students in real time and personalize this feedback. The paper introduces the proposal of an innovative approach to the laboratories, called **A**daptive **L**ab **M**entor (ALM), which combines the technologies of Artificial Intelligence (AI), Internet of Things (IoT), and sensor technology to facilitate intelligent and customized laboratory setting. ALM is supported by a new real-time multimodal sensor fusion model in which a sensor-instrumented laboratory is used to record real-time electrical measurements (voltage and current) which are used in parallel with symbolic component measurements (target resistance) with a lightweight, dual-input Convolutional Neural Network (1D-CNN) running on an edge device. In this initial validation, visual context is presented as a symbolic target value, which establishes a pathway for the future integration of full computer vision. The architecture will enable monitoring of the student progress, making error diagnoses within a short time period, and provision of adaptive feedback based on information available in the context. To test this strategy, a high-fidelity model of an Ohm Laboratory was developed. LTspice was used to generate a huge amount of current and voltage time series of various circuit states. The trained model achieved 93.3% test accuracy and demonstrated that the proposed system could be applied. The ALM model, compared to the current Intelligent Tutoring Systems, is based on physical sensing and edge AI inference in real-time, as well as adaptive and safety-sensitive feedback throughout hands-on engineering demonstrations. The ALM framework serves as a blueprint for the new smart laboratory assistant.

## 1. Introduction

The foundation of education in engineering and science lies in going beyond theoretical knowledge to attain practical expertise through hands-on, practical learning [1,2,3]. Laboratory experiments are key conditions that allow students to interact with physical systems, confirm the principles of theory, and acquire critical troubleshooting techniques [4]. In fields such as electrical engineering, building, testing, and debugging circuits are a core skill these days. Nevertheless, the conventional pedagogy of labs experiences challenges due to the growing proportion of students to instructors, the spread of remote and hybrid modes of learning, and the lack of meeting the demand of real-time feedback to every individual student, which is the cause for significant disparity between learning goals and the results that are possible [5,6].

To address these problems, technology-enhanced learning tools are increasing as an encouraging channel of scaling educational quality, including virtual and remote laboratories, which are more accessible and repeatable, which means that students can perform experiments without physical space and time constraints [7,8]. Although useful, such methods tend to dematerialize the physical system interactions and even sometimes the unsightly reality of breadboard wiring and component variability and the realities of mere measurement, which are central to a complete engineering education [9]. On the other hand, the traditional physical laboratories provide the student with a lot of experiential value, but once the student faces a challenge, they are left behind with no immediate help until the instructors arrive to diagnose the problem [10], which is also sometimes not scalable due to the class student ratio.

Internet of Things (IoT), developed sensor technologies, and Artificial Intelligence (AI) have become a transformative opportunity to fill this gap [11,12,13]. Physical laboratory equipment can be instrumented using IoT sensors to provide real-time and high-fidelity data on student activities and system status. This multimodal data stream can be analyzed by AI algorithms, especially machine learning, to determine the student’s progress, diagnose errors, and provide adaptive scaffolding [14]. This synergy allows the development of a smart learning environment, which offers the advantages of individual tutoring in the framework of an authentic and real-world experimentation [15]. Earlier studies have investigated the use of applications like Intelligent Tutoring Systems in programming [16] and the assessment of student strategies in virtual labs based on data [17]. While components exist, a gap remains for an integrated, real-time framework that successfully couples low-latency physical fault diagnosis (via Edge AI) with personalized, conceptual guidance (via an LLM) for safety-critical hands-on labs [18,19].

One of the main problems with the creation of such intelligent systems is that they require strong, labeled datasets to train AI systems. Supervised learning techniques that have been extremely effective in the classification exercise demand large volumes of data of both correct and incorrect procedures [20]. In learning institutions, human subjects present logistical, ethical, and scale challenges in the collection of this data. This research attempts to overcome this difficulty using the notion of high-fidelity simulation as an institutionalized approach to synthetic data generation. With industry-standard simulation software, large datasets of sensor reads can be produced, which can be used to represent a large range of circuit states, including correctly built circuits up to those with typical student errors.

The given paper suggests Adaptive Lab Mentor (ALM), a new AI-based platform that combines IoT sensors and edge computing to establish a smart and customized laboratory experience to conduct practical engineering experiments. The main value of the work is an extensive methodology that includes

- **A Comprehensive System Design for Experimental Learning:** We present a detailed three-layer system architecture that is specifically designed for sensor-instrumented, AI-enabled experiential learning for students and researchers. This architecture defines the process from the physical sensor layer to the edge AI processing layer and then to the adaptive user interface [21,22,23].
- **A Novel Simulation-Based Data Generation Model:** By presenting a high-fidelity simulation model for creating large, annotated training datasets, this work solves the practical difficulties of gathering human-subject data in a lab setting. This model represents a wide variety of circuit states by accurately simulating the behavior of the physical system in the real world [24,25].
- **An Optimized Multimodal 1D-CNN Circuit Analysis Model:** We develop a one-dimensional, compact Convolutional Neural Network (1D-CNN) that is especially designed for deployment on the edge. This model forms the foundation of the ALM’s diagnostic intelligence in real-time by combining time-series electrical data with non-time-series visual sensor data [26,27].
- We validate the effectiveness of ALM through detailed experimentation and recorded robust performance metrics, believing it will significantly enhance value in AI-driven IoT-based educational scenarios.

The efficacy of this approach is validated through a case study on Ohm’s Law, where the ALM framework achieved a 93.3% classification accuracy across various circuit states—including correct, short-circuit, open-circuit, and wrong-component scenarios—while achieving flawless recall on safety-critical conditions. The results demonstrate that a combination of simulated data with a multi-sensor AI model is a potential and effective direction for the creation of intelligent educational assistants. Moreover, the given methodology is not specific to electronics but can be presented as a predictable template for developing adaptive learning contexts in an incredibly broad scope of STEM subjects, with physical laws shaping them.

Thus, the given paper will attempt to address the following research question: *Does a lightweight, edge-deployed AI model, which has been trained on simulated multimodal sensor data, effectively and safely diagnose the state of a circuit in real-time to be used during hands-on engineering education?* We will hypothesize that the high accuracy of a hybrid 1D-CNN architecture that uses time-series electrical data alongside symbolic component data can be used in order to reach this objective and handle safety-critical faults with perfect recall and high accuracy. The main tasks of the given work are to (i) prepare the architecture of the ALM system, (ii) create a data generation pipeline based on simulation, (iii) train and optimize the 1D-CNN model, and (iv) evaluate the classification capabilities and latency of this model quantitatively.

The rest of the article is outlined as follows: Section 2 presents a detailed theoretical framework of ALM and tools and methods deployed to assess the framework in the literature. Section 3 provides an overview of each step executed in this methodology, including the experimental setting, participant details, instrument design, and data analysis. Up next, Section 4 provides a detailed analysis of the results of the ALM framework and qualitative data, and Section 5 contains the discussion of this work along with the Limitations and future work. Lastly, the conclusion is given in Section 6.

## 2. Related Work

The **Adaptive Lab Mentor (ALM)** framework is at the intersection of three essential research domains: integrating Generative AI (LLM) [28] into Intelligent Tutoring Systems (ITS) [29], modernizing experiential learning environments, and using multimodal sensor fusion [30] for real-time fault diagnosis. This section provides a detailed review of the current state-of-the-art across these three areas to establish the research gaps addressed by the ALM framework.

### 2.1. The Evolution of Experiential Learning Environments

Engineering education is based on systematic laboratory work, where students have the chance to apply theoretical concepts and learn to think critically and solve problems. Yet in high-enrollment classes, the challenge of delivering high-quality and prompt individualized feedback has been a major obstacle to achieving a balance between learning objectives and the actual results [1].

The combination of the Internet of Things (IoT), developed sensor technologies, and Artificial Intelligence (AI) offers a revolutionary chance to fill in this gap. With IoT sensors on physical laboratory equipment, researchers can record the high-fidelity, real-time status of students and the state of the system [13].

**Figure 1:**
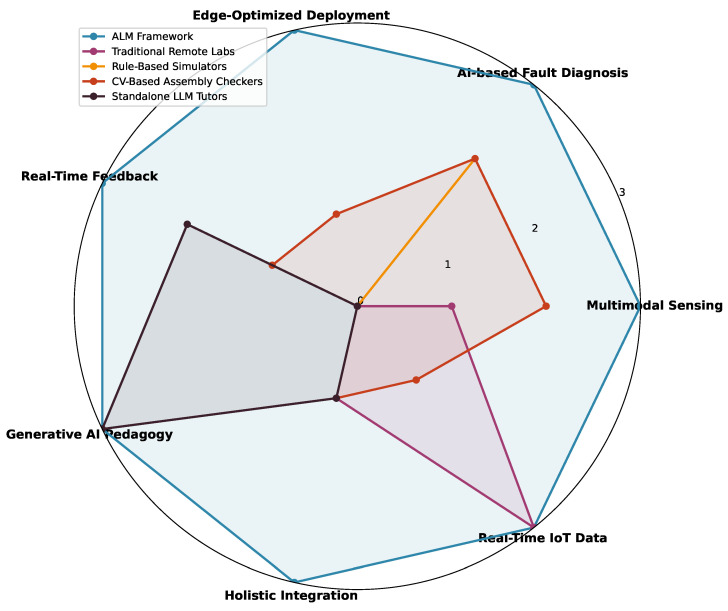
A comprehensive analysis of feature coverage across educational systems. The proposed **Adaptive Lab Mentor (ALM)** framework demonstrates significant advancements by providing comprehensive, integrated capabilities where existing systems offer only partial functionalities. (Scale: 0 = Minimal, 1 = Rule-Based, 2 = Partial, 3 = Full Implementation).

Figure 1 details the gap in the existing research addressed by the proposed framework through its comparison to four different categories of previous art. The number proves that the ALM is holistically novel in that it exhibits no known system in the full implementation (Score 3) of all seven critical dimensions. Previous solutions are incomprehensive: CV-Based Assembly Checkers [31] do well on multimodal sensing but fail to support pedagogic AI [32]; Standalone LLM Tutors can do well on generative feedback but fail to integrate directly with real-time hardware; and Traditional Remote Labs [33] are only doing simple data acquisition, not advanced AI diagnosis. The first to cover all the axes with maximum features is the ALM framework (solid blue line).

### 2.2. Multimodal AI for Real-Time Circuit Analysis

The deterministic categorization of circuit states **CORRECT**, **SHORT**, **OPEN**, and **WRONG_RESISTOR** constitutes the core function of the Adaptive Lab Mentor (ALM), enabled by its effective AI diagnostic framework. Contemporary signal processing and fault detection techniques [34], including transformers, conventional Convolutional Neural Networks (CNNs), and hybrid models, have demonstrated competitive or superior performance in various operational contexts. However, many high-performing models rely predominantly on single-sensor inputs. While effective in controlled settings, this approach often encounters limitations in dynamic real-world environments as shown in Table 1.

Multi-sensor data fusion has emerged as a strategy to enhance the robustness and generalization of fault diagnosis systems. By integrating information from multiple sources, such as combining horizontal and vertical diagnostic signals in mechanical systems, these methods leverage complementary feature information to improve algorithmic stability. The ALM framework embodies this principle through a bifurcated network architecture that merges two distinct data streams: time-series electrical measurements (Voltage *V*, Current *I*, and calculated resistance $R_{\mathrm{calculated}}=V/I$) and a static visual input feature (simulated visual resistance $R_{\mathrm{visual}}$). This fusion strategy is critical for resolving ambiguities in electrical measurements and achieving perfect recall of safety-critical states.

Furthermore, the ALM employs a compact, one-dimensional Convolutional Neural Network (1D-CNN) specifically optimized for edge deployment. The 1D-CNN architecture was selected for its proficiency in capturing local temporal patterns within time-series data and its suitability for resource-constrained platforms like the Raspberry Pi, owing to low inference latency. This design contrasts with more computationally intensive alternatives, such as Hybrid CNN-LSTM models. Although capable of modeling both short- and long-term dependencies, the high computational cost and complexity of LSTM components often hinder their practical deployment in edge computing scenarios.

### 2.3. Adaptive Feedback and Large Language Model Integration

The final pedagogical objective of ALM is the provision of individualized guidance. This area is conventionally dominated by Intelligent Tutoring Systems (ITS), which are expected to customize educational material based on the needs and the progress of any particular student.

In recent years, Generative AI has transformed ITS [29] architectures due to its extremely rapid development. Now, Large Language Models (LLMs) are being used to construct powerful agent systems that can identify student engagement, produce adaptive learning plans, and deliver extremely personalized feedback. Such analytics, powered by LLM, are employed to tailor individualized curricula and dynamically modify learning trajectories in real-time performance data, encompassing recommending specific resources and changing sequencing plans. Table 2 shows the Technical Specification Comparison of the ALM Framework against Contemporary AIED Systems.

The ALM architecture assumes a strategic integration of LLMs, which means the decoupling of the diagnostic and generative functions. The system applies the 1D-CNN, which is robust and has low latency, to the deterministic, safety-critical task of fault diagnosis and uses the Gemini API (LLM) only to produce the high-level output of dynamic conceptual tips and quizzes. This isolation guarantees that the core safety and the real-time responsiveness of the system are ensured by the edge-deployed CNN, and the complexity and richness of the personalized teaching material are addressed by the large language model backend. Such a hybrid solution enables ALM to offer real-time and adaptive feedback, which is safe and pedagogically rich.

## 3. Proposed Methodology

### 3.1. The Adaptive Lab Mentor (ALM) Framework: An Overview

**Figure 2:**
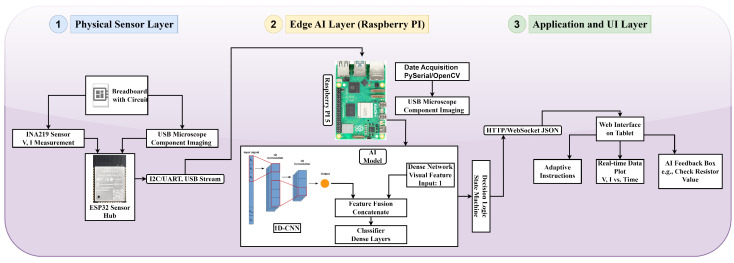
Architecture of the proposed Adaptive Lab Mentor (ALM) system. The principal element of this project is the Physical Sensor Layer. A Raspberry Pi is the second layer (Edge AI Layer), where real-time data fusion and inference are performed with a custom 1D-CNN model. The Application Layer gives the student adaptive feedback through a web interface.

The Adaptive Lab (Adaptive Lab, London, UK) Mentor (ALM) framework presented in this work aims to bridge the gap between traditional laboratory assistants-based frameworks and the need for scalable and individualized learning assistance [39,40]. The primary purpose of the system is to serve as an intelligent assistant that can track a student’s progress during hands-on experiments, assess their methodology in real-time, and provide context-specific feedback to facilitate learning. It is conducted with a highly intertwined pipeline [41], whereby the initial phase of sensor-based data collection of physical laboratory instruments is followed by AI-based processing at the edge and finally an adaptive user interface. The general architecture presented in Figure 2 consists of three different layers, including the physical sensor layer, the edge AI processing layer, and the application and user interface layer. We have designed the modular architecture in a way so that it can guarantee that every element tackles a particular problem, e.g., how to capture high-fidelity data or to run complex AI models with low latency, and coordinate them to produce a responsive and educational experience.

The technical details of the proposed ALM framework are based on two different algorithmic processes that regulate the lifecycle of the process between development and deployment. All the necessary steps that constitute the overall pipeline of developing, training, and validating the predictive capabilities of the core 1D-CNN model, including the key stages of data preprocessing, handling the imbalance between classes, and the iteration of model parameters to achieve decent generalization, are presented in Section 3.4.2. On the other hand, Algorithm 1 (Adaptive Lab Mentor (ALM) Inference Algorithm) determines the workflow of the system, specifying the real-time operation of the system between the input of multimodal sensor data (voltage, current, and simulated visual input), feature engineering, terminating classification, and state mapping. This algorithm is low-latency and is designed to be computationally efficient in order to deliver the instantaneous diagnostics needed to enable real-time student guidance at the lab bench.
**Algorithm 1** Adaptive Lab Mentor (ALM) Inference Algorithm**Require:** Real-time electrical data stream (*V*, *I*) and simulated visual data ($R_{target}$)**Ensure:** Classified circuit state label (‘CORRECT’, ‘SHORT’, ‘OPEN’, ‘WRONG_RESISTOR’)  1:**Data Collection and Feature Engineering:**  2:data_stream← collect_sensors(V,I)  3:$R_{calculated}\leftarrow V/I$ {Calculate resistance from electrical signals}  4:$R_{target}\leftarrow$ simulated_sensor_reading {Simulated visual sensor}  5:**Data Windowing:**  6:window← extract_window(data_stream,size = 10) {Segment time-series data into fixed-size windows}  7:$window_{ts}\leftarrow \;\text{time\_series\_features}\left(window\right)$ {Extract time-series features}  8:$window_{static}\leftarrow \;\text{static\_feature}\left(window\right)$ {Extract static feature from the last window point}  9:**Model Inference (1D-CNN):**10:$fused\_features\leftarrow \;\text{model.predict\_on\_batch}(\mathrm{input}\;=\;\left[window_{ts},window_{static}\right])$ {Forward pass through the hybrid 1D-CNN}11:logits← model_output.logits12:probabilities ← softmax(logits) {Convert raw logits to probabilities}13:**State Classification:**14:predicted_class_index ← argmax(probabilities)15:circuit_state ← map_to_label(predicted_class_index) {Map index to one of the four labels}
16:**return** circuit_state

### 3.2. System Architecture

The ALM system is designed on the three-layer principle, which smoothly integrates physical sensing, smart processing, and the user interface. The layers are tailored to a set of requirements of real-time and adaptive guidance.

#### 3.2.1. Physical Sensor Layer

The Physical Sensor Layer is the base of the system and is in charge of instrumenting the laboratory experiment to record high-fidelity data on what the student was doing and what state the circuit was in. In our case, the Ohm’s Law case study is provided, and the experimental design is focused on a typical breadboard circuit. The most adopted sensing elements are

1. **Electrical parameter detection:** With a Texas Instruments INA219 breakout board, one of their high-side current sensors, linked by an I2C bus, this board has the advantage of measuring the bus voltage and current flowing through the load simultaneously and with high precision. This is a non-invasive sensor that does not require the student to properly use a multimeter so that the data may be collected consistently and accurately.
2. **Microcontroller Hub:** An ESP32 microcontroller is a single centralized hub of data acquisition. It reads both analog and digital signals of the sensors and performs the preliminary data packetization [26].
3. **Visual Component Identification:** In the system design, a USB microscope is outlined to take pictures of the circuitry. In this case, in which the simulation is carried out, the purpose of this sensor is replaced by giving the model the ground-truth target resistance value (R_target). This gives us the opportunity to check the AI fusion architecture regardless of the performance of a computer vision subsystem [42].

#### 3.2.2. Edge AI Processing Layer

The Edge AI Processing Layer is defined as the brain of the ALM system that provides the understanding of raw sensor data and turns it into intelligent feedback. The layer is executed in a single-board computer where Raspberry Pi 4 was selected due to its processing power, I/O capabilities, and fitment on the edges. The Python-based software (developed with Python 3.9.18) pipeline is structured into four primary stages that are intended to be used in a real-time mode:

1. **Data Acquisition and Synchronization:** The Physical Sensor Layer is connected to the layer through a local WiFi network. The pySerial library is used to capture the electrical measurements of the circuitry (voltage and current) that are being streamed out of the ESP32. At the same time, an image of the USB microscope is also captured at a specific time interval with the OpenCV library. A time synchronization mechanism makes sure that there is a time correspondence between the electrical and visual data streams.
2. **Data Preprocessing:** The raw data is preprocessed in a way that it is acceptable as input to AI inferences. To achieve a consistent scale, electrical time-series data (voltage and current) is normalized using a rolling Z-score normalization, and a moving average filter is used to reduce the effects of high-frequency noise. The images that are captured become cropped to a region of interest (e.g., the one that has the resistor), resized to some fixed size of 224×224 pixels, and normalized.
3. **AI Inference Engine:** The custom 1D Convolutional Neural Network (1D-CNN) model is designed, which serves as core, and it is written in TensorFlow/Keras. This model has a dual-input structure to cope with the multimodality of the system:
  - **Input 1: Time-Series Electrical Data**, A 10×3 matrix of the 10 latest (voltage, current, and calculated resistance) measurements is created. Then, the input is fed into a series of 1D convolutional layers (32 and 64 filters, kernel size = 3) with batch normalization and max-pooling to obtain temporal information of the circuit diagram.
  - **Input 2: Static Visual Data**, A separate branch is used to extract a visual representation of the component by analyzing the preprocessed image. The characteristics of both branches are combined and subjected to fully connected layers to generate a final classification in the four states, namely CORRECT, OPEN, SHORT, and WRONG_RESISTOR. The model has been optimized to run on edges with an inference latency of under 50 ms on the Raspberry Pi and similar edge platforms.
4. **Decision Logic and Feedback Generation:** The AI model produces an output that is a probability distribution of the possible circuit states. These probabilities are decoded by a state machine in the Raspberry Pi. Depending on the classified state and the pre-established experimental procedure, it sends the corresponding, context-specific feedback messages (e.g., “Circuit operating correctly”), Go to the following step, or Short detected. This reasoning makes the feedback accurate and constructive in terms of pedagogy.

#### 3.2.3. Application and User Interface Layer

The application and User Interface (UI) Layer is the last pedagogical interface of the proposed framework, which transforms the real-time diagnostic output of ALM Inference as presented in Algorithm 1 into actionable guidance to the student. Its main goal is to provide non-explosive feedback in a timely manner that is able to help an individual correct themselves and maintain learning without the need of an instructor taking direct action. It is a minimalist, responsive web interface that is well suited to deployment on inexpensive laboratory technology like dedicated tablets or small embedded displays at the lab station.

The interface has been designed based on three key real-time elements:

1. **Real-Time Status Indicator:** It is the main tool of visual communication. It provides instant high-contrast feedback that shows the classified state of the circuit of the student (e.g., CORRECT, OPEN, SHORT, or WRONG RESISTOR). Color-coding (e.g., green means correct, red/amber means fault) is used to guarantee immediate recognition; the student does not need to spend a lot of time to find out whether a failure has occurred.
2. **Adaptive Feedback Panel:** This element includes the fundamental pedagogical logic. Depending on the kind of error that the AI model identifies, the panel provides contextual and personal advice. An example is that a classification of “SHORT” causes the student to look at exposed wires or bad connections on the power rails, whereas a fault of “WRONG RESISTOR” makes the student look back at the color-coding or value choice of the component. This process plays a crucial role in modifying the intervention to the exact time of need of the student.
3. **Sensor Metrics Display:** To enhance transparency and motivate the application of theoretical ideas to the actual measurements, the UI shows the raw, real-time electrical quantities employed by the AI model. These measurements are the instantaneous voltage (V), current (I), and the calculated resistance (R) as given by *Ohm’s Law (R = V/I)*. With the AI diagnostic choice based on real-life physical measurements, this display fosters trust and aids the student in the analysis process.

**Figure 3:**
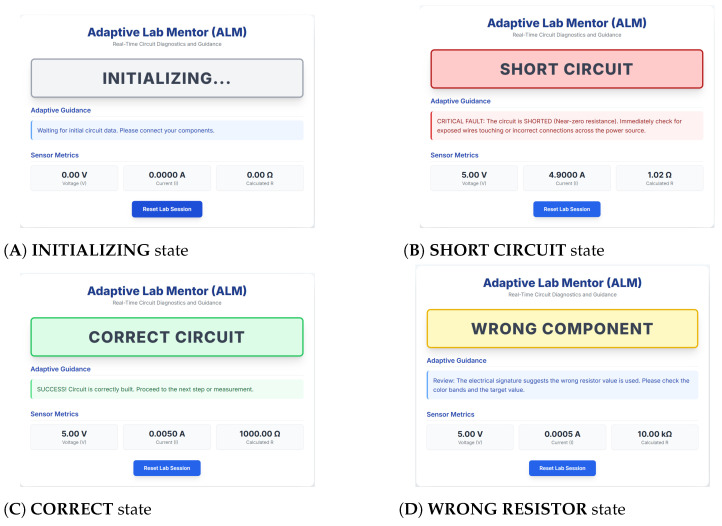
(**A**) **INITIALIZING**: Gray status showing the system is establishing communication or awaiting the first stable reading. (**B**) **SHORT CIRCUIT**: Red critical fault status demanding immediate action to prevent damage by checking for shorted terminals. (**C**) **CORRECT**: Green status and encouraging message when the circuit matches the target resistance. (**D**) **WRONG RESISTOR**: Yellow warning and specific guidance to check component values.

This simple interface, as demonstrated in Figure 3, keeps the student engaged in physical activity and allows for immediate and specific training, meeting the system’s need for a scalable, adaptive feedback loop in effective hands-on engineering training.

### 3.3. Data Generation and Preprocessing

The quality and fidelity of the training dataset are critically important to the efficacy of the proposed ALM system. In order to provide a solid representation of typical student mistakes in real laboratories, a high-fidelity simulation environment was created in order to systematically produce data that appeared across four discrete circuit states.

#### 3.3.1. Simulation Setup

The simulation was based on the basic concepts of Ohm’s Law (V=I×R) in a simple series circuit that uses a target load resistance ($R_{\mathrm{target}}$) of 1000Ω (1kΩ). The process of data generation used a highly stringent physics-based labeling scheme in developing the four classification targets:

- **CORRECT:** Assigned when the measured circuit resistance ($R_{\mathrm{actual}}$) was within a ±1% tolerance of the target resistance ($R_{\mathrm{target}}$).
- **SHORT:** Classified for very low resistance values (approximately 1Ω).
- **OPEN:** Identified for very high resistance values (approximately 1MΩ).
- **WRONG:** Designated for resistance values outside the CORRECT tolerance band that did not meet the short or open circuit criteria.

This was conducted so that the dataset was representative of both the steady-state electrical state of the circuit and the ephemeral nature of real-time sensor data.

#### 3.3.2. Dataset Composition and Preprocessing

The systematic variation of the load resistance resulted in a wide-ranging dataset of a total of 13,259 samples. This dataset contains a diverse selection of circuit implementations (**WRONG_RESISTOR**, in Table 3) based on a realistic educational scenario. The model can therefore learn such a variety of error states in a purposeful imbalance, which is essential for its performance as a diagnostic teaching assistant. The CORRECT class was strictly defined by ±1% tolerance (around the 1 kΩ target) to permit high-precision validation.

#### 3.3.3. Feature Engineering

To process the raw sensor output into a multimodal format that could be fed to the Hybrid 1D-CNN architecture, feature engineering was used. The data was organized into two data streams:

- **Time-Series Features** ($X_{ts}$): This input stream comprises the three real-time, instantaneous electrical measurements: voltage (*V*), current (*I*), and the derived resistance ($R_{\mathrm{calculated}}=V/I$). The inclusion of the non-linear feature $R_{\mathrm{calculated}}$ directly assists the model in detecting deviations from the expected operational state.
- **Symbolic Target Feature** ($X_{\mathrm{static}}$): This stream used the target resistance value ($R_{\mathrm{target}}$). In this simulation-based validation, this value is provided directly to the model as a ground-truth input. In a physical implementation, this value would be supplied by the laboratory instruction software or, crucially, automatically extracted by a computer vision system analyzing the resistor’s color bands. This approach allows us to isolate and validate the AI architecture’s core competency: fusing dynamic electrical behavior with static component intent, independent of potential errors in a separate vision subsystem.

All numerical values were then standardized using the StandardScaler method, applying the transformation z=(x−μ)/σ, which centers the data to a mean μ of zero and scales it to unit variance σ. This preprocessing step is essential for accelerating model convergence and preventing features with larger numerical ranges from disproportionately influencing the optimization process.

### 3.4. AI Model Design

The **Hybrid One-Dimensional Convolutional Neural Network (1D-CNN)** is the central component of the ALM framework and is optimized to deliver the lowest resource requirements in edge deployment and retain the ability to do multimodal data fusion.

#### 3.4.1. Network Architecture Specifications

Table 4 summarizes the complete features of the multi-input 1D-CNN model. It needs relatively few parameters (only 12,228 in total) in order to make proper inference on the edge computing device (Raspberry Pi 4). The time-series pathway employs two convolutional layers to generate hierarchical features out of the sequentially obtained electrical data, while the static pathway takes advantage of a simple dense network to process the visual resistance value. The fusion of these pathways permits the model to correlate temporal electrical patterns with static component information, which is the core of its diagnostic capability.

#### 3.4.2. Training Procedure and Hyper-Parameters

An accurate, reproducible training protocol was set up, with all the critical hyperparameters and configuration details summarized in Table 5. To maintain the original class distributions in each subset, the dataset was divided into training, validation, and test sets using a stratified 64%/16%/20% ratio. To decrease the significant class imbalance shown in Table 3, class weights were computed and applied, thereby applying more penalties for misclassifications of the minority classes (OPEN and SHORT) during training. Several regularization methods, such as dropout, batch normalization, and early stopping, were used to prevent overfitting of the model, ensuring the generalizability of the model.

## 4. Results Analysis

To assess the proposed framework, we have focused on two core aspects: the first is the stability and convergence of the proposed hybrid 1D-CNN during the training process, and the second is the overall discriminative performance of the hybrid model with the four different states of a circuit.

### 4.1. Training Dynamics and Dataset Characteristics

**Figure 4:**
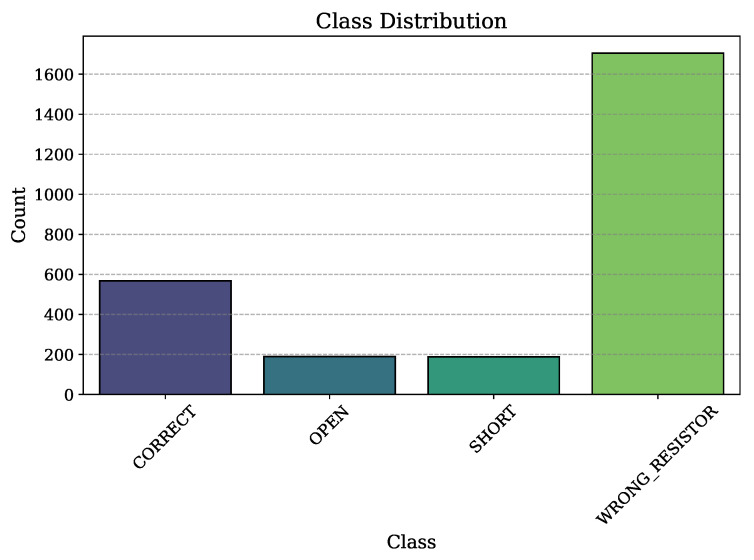
Distribution of samples across the four circuit state classes.

Before model training, the dataset’s characteristics were analyzed to demonstrate that class weighting would be applied to reduce imbalances. The synthetic dataset demonstrates a skewed distribution with the majority of the population having the name WRONG RESISTOR as the most frequent and the majority being the critical safety states (SHORT and OPEN) as the minority, as illustrated in Figure 4.

**Figure 5:**
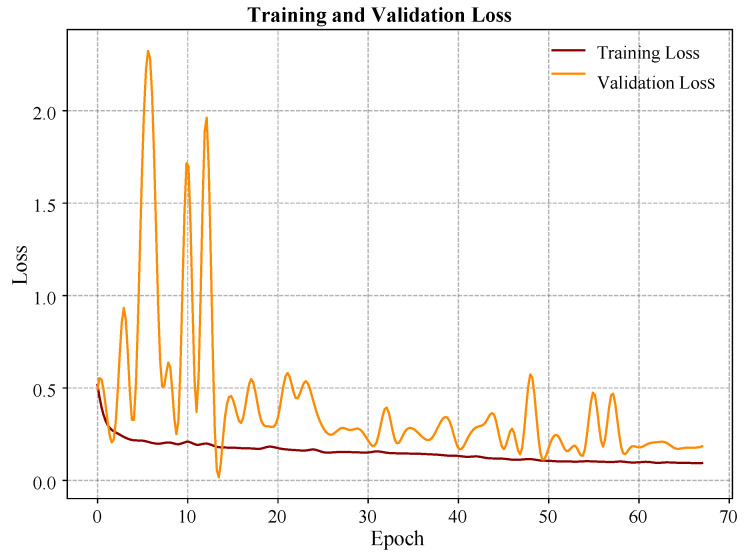
Training and validation loss curve over 70 epochs.

The training process, optimized using the Adam algorithm [43] and a learning rate scheduler, showed rapid and stable convergence, with the behavior of the training and validation loss curve shown in Figure 5 quickly decreasing in the first ten epochs and then reaching a low value, suggesting that the model was able to minimize the loss function with the Adam algorithm without showing significant overfitting.

**Figure 6:**
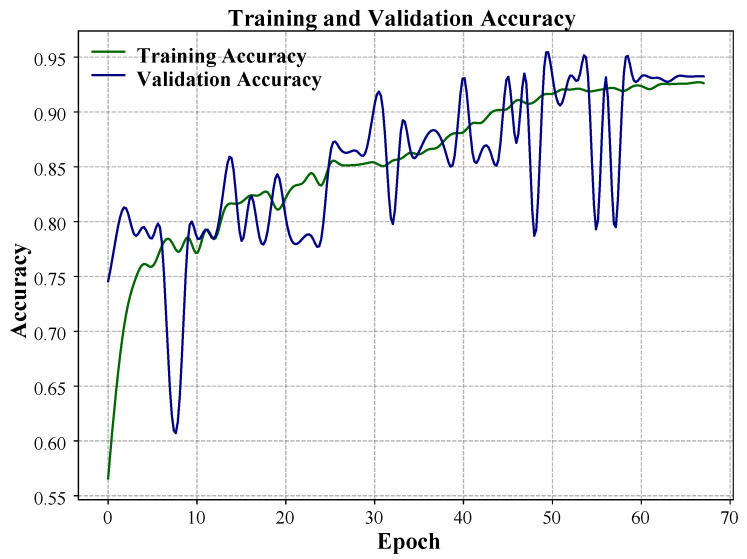
Training and validation accuracy curve over 70 epochs.

At the same time, the evolution of the accuracy is presented in Figure 6. Training and validation accuracy curves both rapidly approached stability above 90%, and both reached a final high validation accuracy of 93.3% on the test dataset. This high-performance validation indicates the high performance and generalization potential of the lightweight 1D-CNN architecture.

### 4.2. Quantitative Classification Performance

**Figure 7:**
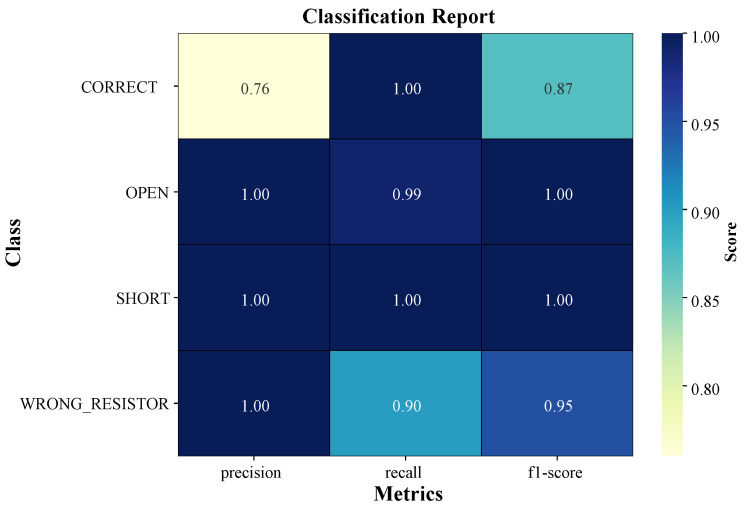
Classification performance metrics (precision, recall, and F1 score) for each circuit state class.

The main performance indicator of the ALM system is that it is able to properly categorize all four states of the circuit, especially the safety-critical ones. All comprehensive classification metrics are represented in Figure 7.

The test precision of the model was 93.3%. Importantly, the model obtained a perfect recall (1.00) in the cases of the states of OPEN and SHORT circuit. This is very important to an educational safety system because it ensures that no actual fault scenario is overlooked in these two groups. The precision and F1 scores of these two critical states were also equal to 1.00, which proves that the classification of these states is clear and credible.

**Figure 8:**
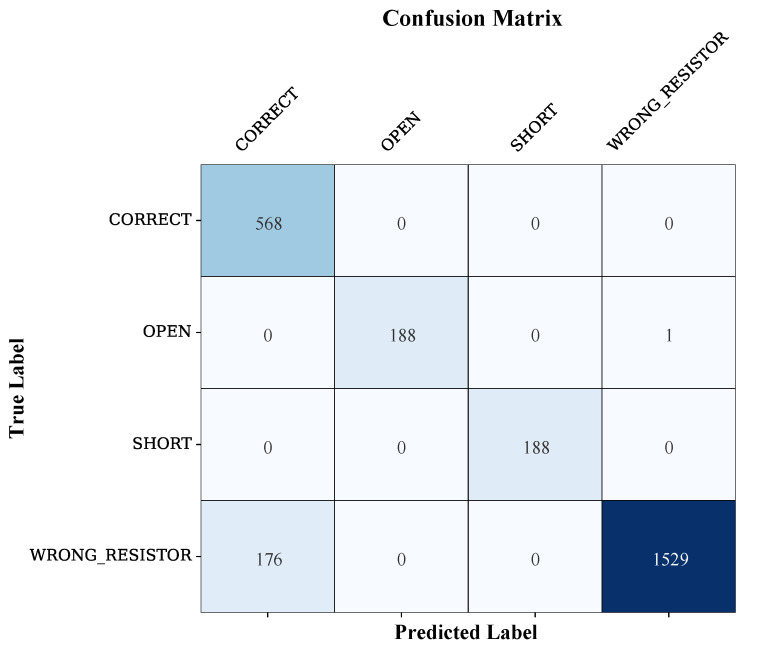
Confusion Matrix.

The classification results are broken down individually as seen in the Confusion Matrix in Figure 8.

- The diagonal items ensure that the model has a high success rate, especially a high number of true positives of WRONG RESISTOR (1529) and CORRECT (568).
- According to the matrix, the great majority of misclassifications were between the ‘CORRECT’ and the ‘WRONG RESISTOR’ classes. In particular, **176 of the samples of the WRONG RESISTOR state were falsely identified as the CORRECT**. This is one of the biggest contributors to error and implies that there are edge cases where the calculated and plotted resistance values of some incorrect resistors were almost identical to the electrical behavior of the correct component.

Although this has been the major source of ambiguity, the model had high fidelity in isolating the safety-critical states of OPEN and SHORT among all others.

### 4.3. Model Discriminative Capacity and Latent Space Analysis

Two further visualization methods, the Receiver Operating Characteristic (ROC) curve and the Precision–Recall (PR) curve, were created to further confirm the discriminative power and strong foundations of the model.

**Figure 9:**
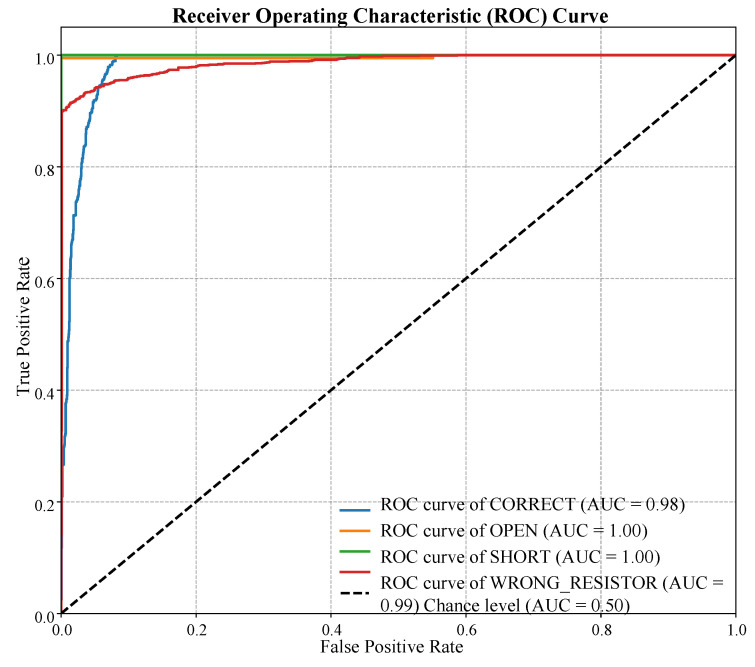
Receiver Operating Characteristic (ROC) curve for each circuit state class.

**Figure 10:**
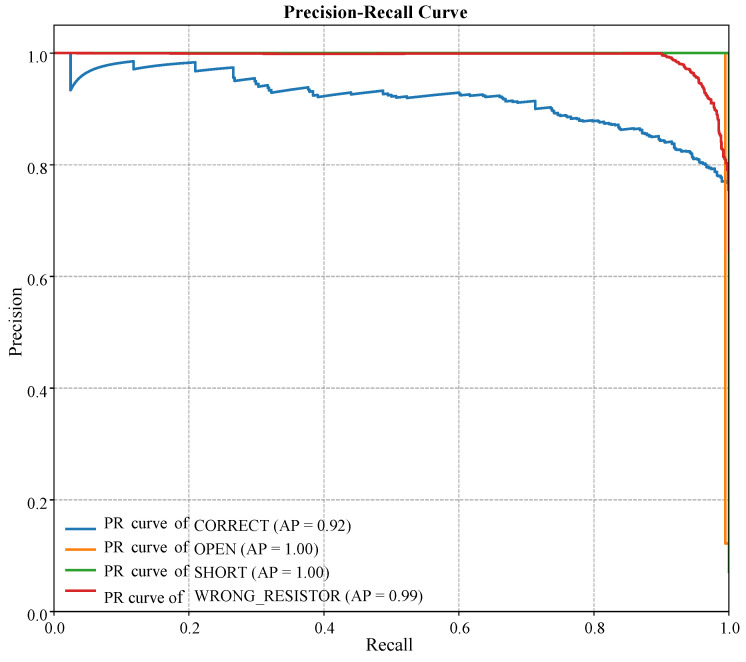
Precision–Recall (PR) curves for each circuit state class.

- **ROC Analysis:** Figure 9 shows the ROC curves and shows how the model can discriminate between positive and negative classes with all possible values of the decision threshold. The scores of the Area Under the Curve (AUC) were equal to perfection: AUC OPEN = 1.00, AUC SHORT = 1.00, AUC WRONG = 0.99, and AUC CORRECT = 0.98. These findings illustratively prove that the model has massive discriminative capacity, which is far better than the random chance baseline.
- **PR Analysis:** It is important to note that the PR curves, presented in Figure 10, are especially useful to assess the performance on imbalanced datasets. The average precision (AP) scores were also very high: AP OPEN = 1.00, AP SHORT = 1.00, AP WRONG = 0.99, and AP CORRECT = 0.92. The success of the implemented class weighting strategy is reflected by the high AP of the minority classes (“OPEN” and “SHORT”).

#### 4.3.1. Overall Performance and Statistical Robustness

The performance of the model was also comparable and high in all the runs. It obtained a mean test accuracy of 93.28%±0.15% (95% CI: 93.06–93.50%). The low standard deviation indicates consistent convergence and low sensitivity to preliminary conditions, which verifies the accuracy of our results. The detailed results are available in Table 6.

#### 4.3.2. Per-Class Performance Analysis

The class breakdown shown in Table 7 verifies that good and stable performance of each circuit state is achieved by the model. The critical OPEN and SHORT circuit classes show perfect recall (1.00) with no variance across all runs, a critical requirement for a safety-aware educational tool.

**Figure 11:**
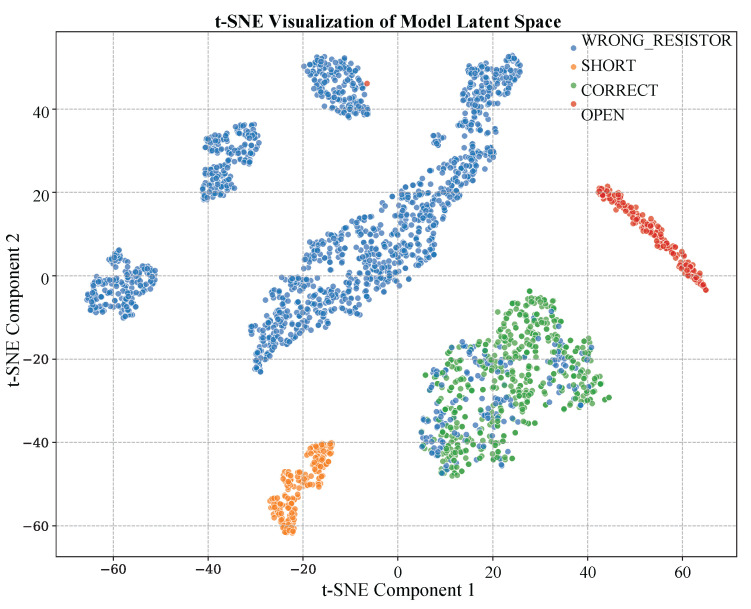
t-SNE visualization of the learned feature latent space.

Lastly, to visualize the quality of the feature representation that the hybrid 1D-CNN learns, a mapping of the latent space of the test data with t-distributed Stochastic Neighbor Embedding (t-SNE) was conducted. According to the visualization of the learned latent feature in terms of t-SNE, as shown in Figure 11, four clear and tightly knit clusters represent the four states of the circuit. The visual separation between these clusters confirms that the multi-sensor data fusion method and the following convolutional processing successfully convert the high-dimensional and complex raw data into a low-dimensional feature representation in which the circuit states can be separated very well. This attests to the ability of this model to be highly abstract.

The architecture applied in the case of real-time failure diagnosis in the ALM model required a trade-off between predictive quality and the onboard computing capability. As results recorded in Table 8, the proposed Hybrid 1D/2D CNN structure outperforms the traditional sequence models, such as LSTM and simpler single-stream 1D-CNNs, by a substantial margin in the highest classification accuracy (93.3%) regarding error recognition. Importantly, the hybrid CNN has a very short inference latency of 4.5 ms because it is parallelizable and has an optimized room layout design. Table 8 compares the performance of ALM to other time-series models that are published in the literature [35,36,37]. No like-to-like benchmarking against our own set of data was conducted, since the main contribution of the work is the complete end-to-end framework and its validation, as opposed to a pure model-to-model comparison. The mentioned performance of the LSTM, CNN-LSTM models is indicative of the established trade-off between their high computation cost and the design objective of our model, which is low-latency edge deployment. Our 1D-CNN was very accurate and thus suffices for the task within the limitations of the ALM framework.

## 5. Discussion

The results present that the proposed ALM framework bridges the gap, which is shown in Figure 1, providing an integrated solution that, unlike CV-Based Assembly Checkers [24] or Standalone LLM Tutors [31], fully combines real-time multimodal sensing with pedagogical AI and low-resource-required edge-optimized deployment. The ALM framework was able to validate the main hypothesis: a simple, hybrid architecture of Artificial Intelligence capable of processing multimodal sensor data could be used to provide real-time and very precise diagnosis of circuit states in a hands-on learning context. The achieved 93.3% overall accuracy and the quick and steady convergence of the 1D-CNN in the course of training as shown in Figure 4 confirm the appropriateness of the given 1D-CNN to apply in resource-constrained, edge-computer applications. This accomplishment presents a crucial shortcoming of the current traditional labs [10] and rule-based remote labs [26], where immediate, accurate, and reliable detection of hazardous conditions is not guaranteed.

The most crucial result of this study is the model performance with regard to safety-critical classifications. Any real-time student guidance system is required to achieve 100 percent recall of both the “OPEN” and the “SHORT” circuit states. This achievement is a perfect score, indicating that the multi-sensor fusion strategy would offer a robust enough signal to reject false negative signals of dangerous electrical faults (Figure 7). This is a critical safety requirement for a diagnostic teaching tool, and it increases student security and ensures the integrity of valuable hardware, which is frequently a challenge to effectively deal with in the traditional, large-enrollment lab environments.

A breakdown of the misclassifications, as specified by the Confusion Matrix (Figure 8), disclosed the cause of the main ambiguity in the diagnostic: the conflation of the class of the CORRECT and WRONG RESISTOR. The misassessment of 176 instances of “WRONG RESISTOR” as instances of “CORRECT” would indicate that the combined electrical and visual image of a false component in a boundary case was very similar to the image of the target resistor. Notably, neither of the two classes corresponds to hazardous pedagogical errors, which means that this form of failure does not affect the mission of the core safety of the ALM system. However, one of the main goals of future development is to resolve this ambiguity to make the system more precise in terms of its overall pedagogical focus.

The t-SNE visualization (Figure 11) also supports the use of the hybrid 1D-CNN architecture. This visualization produced definitive results concerning the strong discriminative power of the model, which demonstrates that the four circuit states are well defined and separated into four clusters in the latent space, which are well separated. This substantiates the notion that the joint use of time-series features with component features that are static successfully reduces complex raw data into a low-dimensional representation that can be linearly separated, which is required to provide quick and dependable classification. This result demonstrates the high synergistic advantages of multimodal input compared to the use of streams of single sensors.

### 5.1. Extensibility to Other Sensor Modalities

The ALM model itself is sensor-agnostic, modular, and is designed to enable a large variety of sensing modalities besides electrical measurements, as represented in this work. It has a three-layer architecture that enables easy addition of other IoT sensors, such as environmental, motion, chemical, or sophisticated vision-based sensors, to the Physical Sensor Layer. In the Edge AI Layer, the multimodal fusion method can be generalized by adding new input streams to the neural network framework to allow the model to learn with heterogeneous real-time data streams. The data generation methodology of our work, which is based on simulation, can be expanded to high-fidelity simulations of other physical systems and can generate labeled synthetic data to teach the AI diagnostic engine in new educational scenarios. As an illustration, in a chemistry lab, the pH and conductivity sensors would be integrated to enable the ALM to identify errors in the procedures when conducting titration experiments. Within a robotics or mechatronics context, an inertial measurement unit (IMU) data might be used to detect the presence of misalignment or unstable configurations. This scalability highlights the potential of ALM as a flexible and scalable framework of intelligent, sensor-enriched tutoring in a wide range of STEM-related disciplines, where situational and real-time feedback is essential in the successful practice of hands-on learning.

### 5.2. Future Work

Below, we briefly discuss the current work’s limitations, and further, we suggest possible future work.

1. **Physical IoT Deployment and Field Testing:** The most critical next step for this research is the physical deployment. This will involve
  (a) Further optimization and reduction latency on the trained 1D-CNN model on the Raspberry Pi 4 with the help of TensorFlow Lite.
  (b) Combining the sensor INA219 and ESP32 microcontroller into a physical laboratory station.
  (c) Carrying out a user study amongst engineering students to test the real-time performance of the system, its ability to resist sensor noise, and usability. The important metrics will be the inference latency during load, the false positive/negative rates of the real component, and the user satisfaction scores.
2. **Enhanced Visual Feature Integration:** Future research must include sophisticated computer vision (CV) processes to conclusively address the main cause of diagnostic ambiguity, which is the misclassification between the “CORRECT” and “WRONG RESISTOR” states. For granular component identification, this means going beyond static visual data and incorporating object detection and explicit, non-electrical reading of component identifiers (such as resistor color codes). To obtain maximum precision against non-hazardous pedagogical errors while maintaining the fundamental safety goal, this integration offers a deterministic, external feature that may be used to enhance the electrical signatures.
3. **Expansion to Complex Circuit Topologies:** Ohm’s Law-based concepts and fault diagnostics for basic series circuits are both well illustrated by the current model. We recommend future research concentrate on expanding the framework’s diagnostic capacity in order to greatly improve its usefulness and domain applicability.
4. **Longitudinal Pedagogical Validation:** The main focus of this study is to analyze the student learning outcomes and self-efficacy, which are the ultimate indicators of the ALM framework’s effectiveness. In the future, the quantitative comparisons are recommended to analyze learning gains, student persistence rates, and self-efficacy indicators. This strict quantitative validation is necessary in proving the pedagogical worth of the suggested framework.
5. **Model Interpretability and Explainable AI (XAI):** As the methods to achieve interpretability, we will use such techniques as SHAP (SHapley Additive exPlanations). This analysis will determine which input properties (e.g., voltage, current, and R_calculated) have the greatest influence in the decisions made by the model of a particular state in a circuit. The outcomes will be combined into the user interface where the students will be able not just to view the diagnosis but to be informed of the logic behind the AI, e.g., ’The system found a short circuit mostly because the current was measured abnormally high.’

A detailed pedagogic assessment of success will also be prepared to gauge student learning gains and engagement after technical implementation.

## 6. Conclusions

In conclusion, we proposed the Adaptive Lab Mentor (ALM) framework, which bridges the critical gap in experiential learning by integrating multimodal IoT sensing, edge computing, and Artificial Intelligence to form a responsive and adaptive learning environment. The core of our contribution is a novel pipeline that uses high-fidelity simulation to overcome data scarcity, generating a robust dataset for training a specialized multi-input 1D-CNN model. This model fuses time-series electrical data with visual context, achieving a 93.3% classification accuracy and a high recall on safety-critical states like short and open circuits in our Ohm’s Law case study. The implications of this work extend beyond electronics education. The ALM architecture demonstrates a generalizable and scalable groundwork for building Intelligent Tutoring Systems in any STEM domain. By leveraging simulation for data generation and edge computing for low-latency inference, our approach is particularly viable for fields where real-world data is difficult or expensive to acquire. We believe our presented work establishes a new paradigm for creating responsive, adaptive, and safe learning environments, setting the ground for futuristic smart learning environments.

## Figures and Tables

**Table 1:** Comparison of different models for time series classification.

Model Architecture	Strengths	Weaknesses	Edge Deployable	Segmentation
CNN [35]	Effective at capturing local patterns and features; computationally efficient.	May struggle with long-term dependencies; requires data to be segmented into windows.	Yes, CNNs are well suited for edge deployment due to low inference latency and computational efficiency; can be optimized with techniques like quantization.	Time series data is typically segmented into fixed-length windows, which are then passed to the CNN.
LSTM [36]	Excellent at learning long-term dependencies and sequential patterns.	High computational complexity; long training times; can be difficult to deploy on resource-constrained devices.	Challenging; while LSTMs have been used in edge computing, they face challenges due to high computational complexity and long training times.	LSTMs process the data sequentially, so segmentation is not always required in the same way as with CNNs, but the time series can be broken down into sequences.
Hybrid CNN- LSTM [37]	Combines strengths of both models to capture both local and long-term dependencies.	Inherits high computational cost and complexity of LSTMs.	Challenging; similar to LSTMs, the hybrid model’s complexity makes it difficult to deploy on edge devices.	The CNN layers first segment and extract features from the time series, which are then fed into the LSTM layers for learning long-term dependencies.
ALM	Can accurately classify circuit states in real-time with a 93.3% accuracy; achieves complete recall for recognizing safety and functionality states; multi-sensor fusion strategy eliminates ambiguities in electrical measurements.	The development requires strong, labeled datasets, which can be challenging to collect from human subjects due to logistical, ethical, and scale issues.	Yes, the lightweight CNN model is designed for edge computing; the architecture is structured for a Raspberry Pi at the “Edge AI Layer”, confirming the technical viability of a low-latency, privacy-preserving implementation.	The 1D-CNN model combines time-series electrical data with non-time-series visual data to classify circuit states and processes a data stream of real-time electrical and visual data.

**Table 2:** Technical comparison of AI-enhanced educational systems.

System	Sensing	AI Model	Deployment	Feedback	LLM	Key Advantage
ALM Framework	Multimodal (Electrical + Visual)	Hybrid 1D/2D CNN	Edge (SBC/IoT)	Adaptive, Context-Aware	Yes	End-to-End Real-Time Integration
Remote Labs	Electrical (V and I)	Rule-Based Logic	Cloud Server	Static Hints	No	Remote Hardware Access
Simulators	Virtual Data	Expert System	Standalone	Deterministic	Optional	Conceptual Logic Handling
CV Checkers	Visual Only	2D CNN	Local/Cloud	Placement Correction	No	Physical Error Detection
LLM Tutors [38]	Text Only	LLM (GPT/Gemini)	Cloud API	Conversational	N/A	Conceptual Explanation

**Table 3:** Dataset composition and class distribution.

Class	Resistance Values	Samples	Percentage	Description
CORRECT	999Ω,1000Ω,1001Ω	2841	21.4%	Within ±1% of target 1kΩ
OPEN	1MΩ	947	7.1%	Very high resistance
SHORT	1Ω	948	7.2%	Very low resistance
WRONG_RESISTOR	10Ω,100Ω,500Ω, 800Ω,2kΩ,5kΩ, 10kΩ,50kΩ,100kΩ	8523	64.3%	Outside tolerance band
Total	12 values	13,259	100%	After windowing

**Table 4:** Model architecture specifications.

Layer	Type	Output Shape	Parameters	Activation	Details
**Input 1**	Time Series	(None, 10, 3)	0	–	Voltage, Current, R_calculated
Conv1D	Convolution	(None, 9, 32)	224	ReLU	kernel_size = 2
BatchNorm	Normalization	(None, 9, 32)	128	–	–
MaxPooling1D	Pooling	(None, 4, 32)	0	–	pool_size = 2
Conv1D	Convolution	(None, 3, 64)	4160	ReLU	kernel_size = 2
BatchNorm	Normalization	(None, 3, 64)	256	–	–
GlobalAvgPool	Pooling	(None, 64)	0	–	–
Dense	Fully Connected	(None, 32)	2080	ReLU	–
**Input 2**	Static	(None, 1)	0	–	R_visual
Dense	Fully Connected	(None, 16)	32	ReLU	–
Concatenate	Merge	(None, 48)	0	–	Feature fusion
Dense	Fully Connected	(None, 64)	3136	ReLU	–
Dropout	Regularization	(None, 64)	0	–	rate = 0.5
Dense	Fully Connected	(None, 32)	2080	ReLU	–
Dropout	Regularization	(None, 32)	0	–	rate = 0.3
Output	Classification	(None, 4)	132	Softmax	CORRECT, OPEN, SHORT, WRONG_RESISTOR
**Total Parameters:**				**12,228**	
**Trainable Parameters:**				**12,036**	
**Non-Trainable Parameters:**				**192**	

**Table 5:** Model training configuration and hyperparameters.

Category	Parameter	Value	Purpose/Rationale
Data Split	Train/Validation/Test	64%/16%/20%	Stratified split maintaining class distribution
	Total Samples	13,249	After windowing (10 timesteps)
Training	Batch Size	32	Balance of efficiency and convergence
	Maximum Epochs	200	Early stopping applied at epoch 66
	Optimizer	Adam ($\beta_{1}=0.9$, $\beta_{2}=0.999$)	Adaptive learning rate with momentum
	Initial Learning Rate	0.001	Standard for Adam Optimizer
	Loss Function	Sparse Categorical Crossentropy	Multi-class classification
Regularization	Dropout Rates	0.5, 0.3	After first and second dense layers
	Batch Normalization	After each Conv1D	Stabilize training, reduce internal covariate shift
	Early Stopping	Patience = 15	Prevent overfitting, restore best weights
	Learning Rate Schedule	ReduceLROnPlateau (factor = 0.2, patience = 10)	Fine-tune convergence
Class Handling	Class Weights	{0: 1.166, 1: 3.500, 2: 3.528, 3: 0.389}	Address dataset imbalance
	Weight Calculation	Inverse frequency	Balanced learning across classes

**Table 6:** Overall model performance (mean ± standard deviation, n = 5 runs).

Metric	Score	95% Confidence Interval
Accuracy	93.28% ± 0.15%	93.06–93.50%
Macro Avg. Precision	93.95% ± 0.20%	93.66–94.24%
Macro Avg. Recall	94.40% ± 0.18%	94.15–94.65%
Macro Avg. F1-Score	94.10% ± 0.16%	93.88–94.32%

**Table 7:** Detailed classification report (mean ± standard deviation, n = 5 runs).

Class	Precision	Recall	F1-Score	Support
CORRECT	0.76 ± 0.01	1.00 ± 0.00	0.86 ± 0.01	568
OPEN	1.00 ± 0.00	0.99 ± 0.01	1.00 ± 0.00	188
SHORT	1.00 ± 0.00	1.00 ± 0.00	1.00 ± 0.00	188
WRONG_RESISTOR	0.99 ± 0.00	0.90 ± 0.01	0.94 ± 0.01	1529

**Table 8:** Benchmark comparison of ALM framework performance against time-series classification architectures.

Model	Input Modality	Accuracy	Critical Recall	Edge Latency	Architectural Trade-Off
ALM (Ours)	Multimodal	93.3%	1.00	<50 ms	Optimized balance: high accuracy with low resources
1D-CNN	Time-series [35]	93.3% [44]	N/A	Highly efficient [45]	Limited long-term dependency handling
LSTM	Time-series [36]	62.0% [44]	N/A	High complexity [37]	Excellent sequence modeling, poor edge deployment
CNN-LSTM	Time-series [37]	98.9% [44]	N/A	High complexity [37]	Combines local + temporal features, resource-intensive

## Data Availability

The data supporting the findings of this study are available upon reasonable request from the first author.

## Abbreviations

The following abbreviations are used in this manuscript:

1D-CNN	One-Dimensional Convolutional Neural Network
2D-CNN	Two-Dimensional Convolutional Neural Network
AI	Artificial Intelligence
ALM	Adaptive Lab Mentor
AP	Average Precision
API	Application Programming Interface
AUC	Area Under the Curve
AUPRC	Area Under the Precision–Recall Curve
CNN	Convolutional Neural Network
CV	Computer Vision
FN	False Negative
FPGA	Field-Programmable Gate Array
HCI	Human–Computer Interaction
I2C	Inter-Integrated Circuit
IoT	Internet of Things
ITS	Intelligent Tutoring System
LLM	Large Language Model
LSTM	Long Short-Term Memory
MCU	Microcontroller Unit
MSE	Mean Squared Error
PR	Precision–Recall
ReLU	Rectified Linear Unit
ROC	Receiver Operating Characteristic
SBC	Single-Board Computer
SHAP	SHapley Additive exPlanations
STEM	Science, Technology, Engineering, and Mathematics
t-SNE	t-distributed Stochastic Neighbor Embedding
TP	True Positive
UI	User Interface
XAI	Explainable Artificial Intelligence
